# Supplementary material for: Juvenile influenza can impair myelin development and adult behavior through chemokine signaling in mice
Source: bioRxiv. 2026 Aug 13:2026.08.08.743593. Preprint. [Version 1] doi: 10.64898/2026.08.08.743593 (PMC13640621; doi:10.64898/2026.08.08.743593)
Supplement: Supplementary file 1 [file NIHPP2026.08.08.743593v1-supplement-1.pdf]

bioRxiv preprint doi: https://doi.org/10.64898/2026.08.08.743593; this version posted August 13, 2026. The copyright holder for this preprint (which was not certified by peer review) is the author/funder, who has granted bioRxiv a license to display the preprint in perpetuity. It is made available under a CC-BY 4.0 International license.

Figure S1

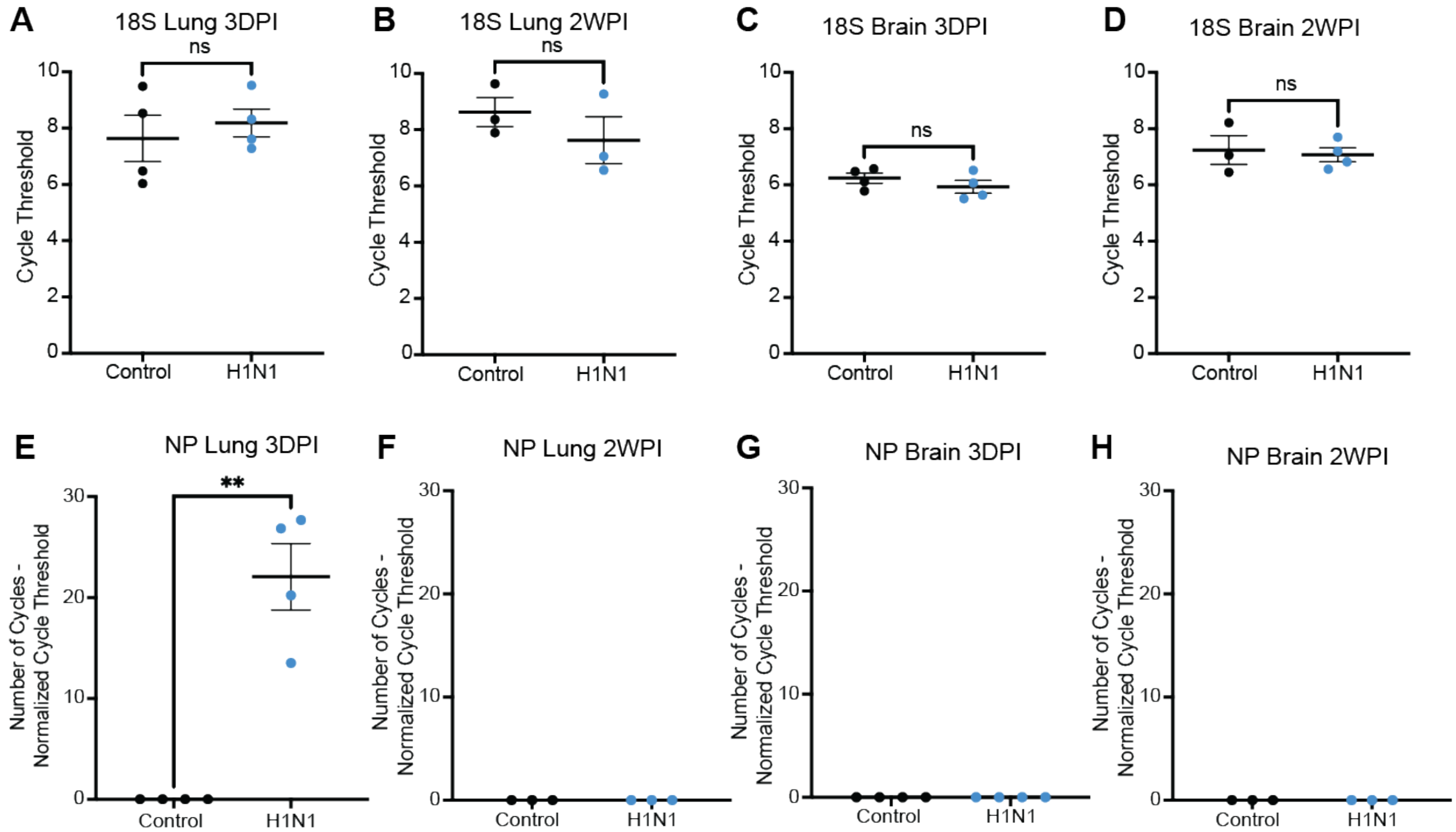

bioRxiv preprint doi: https://doi.org/10.64898/2026.08.08.743593; this version posted August 13, 2026. The copyright holder for this preprint (which was not certified by peer review) is the author/funder, who has granted bioRxiv a license to display the preprint in perpetuity. It is made available under a CC-BY 4.0 International license.

Figure S1. H1N1 virus is confined to the lung and cleared by two weeks, related to **Figure 1**.

(A-D) Quantification of cycle threshold (CT) of 18S in the (A and B) lung or (C and D) brain at (A and C) 3 days (DPI) or (B and D) 2 weeks post-infection (WPI).

(E-H) Quantification of influenza virus nucleoprotein (NP) normalized CT in the (E and F) lung or (G and H) brain at (E and G) 3 days or (F and H) 2 weeks post-infection.

Data shown as mean ± SEM; each dot represents an individual mouse. ns: $p > 0.05$, ** $p < 0.01$, analyzed via unpaired two-tailed t-test.

bioRxiv preprint doi: https://doi.org/10.64898/2026.08.08.743593; this version posted August 13, 2026. The copyright holder for this preprint (which was not certified by peer review) is the author/funder, who has granted bioRxiv a license to display the preprint in perpetuity. It is made available under a CC-BY 4.0 International license.

Figure S2

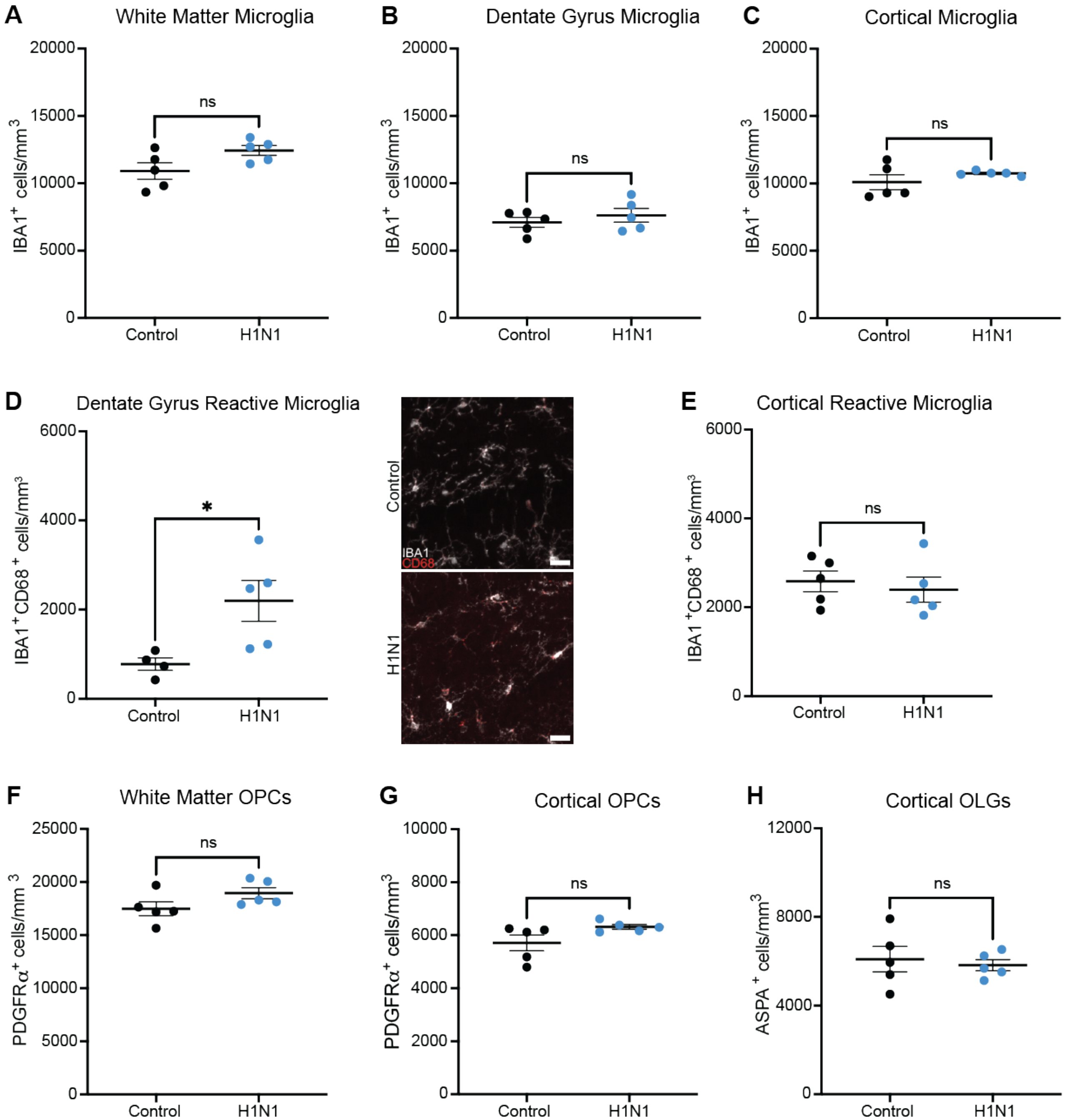

bioRxiv preprint doi: https://doi.org/10.64898/2026.08.08.743593; this version posted August 13, 2026. The copyright holder for this preprint (which was not certified by peer review) is the author/funder, who has granted bioRxiv a license to display the preprint in perpetuity. It is made available under a CC-BY 4.0 International license.

Figure S2. Glial cell counts 7 days post-infection in the white matter, dentate gyrus, and cortex, related to **Figure 1**.

(A) Quantification of white matter microglia (IBA1$^+$) 7 days post-infection in the cingulum. *n* = 5 mice per group.

(B) Quantification of white matter microglia (IBA1$^+$) 7 days post-infection in the hilus of the dentate gyrus. *n* = 5 mice per group.

(C) Quantification of gray matter microglia (IBA1$^+$) 7 days post-infection in the secondary motor cortex (M2). *n* = 5 mice per group.

(D) Quantification and representative confocal micrographs of white matter reactive microglia (IBA1$^+$CD68$^+$) 7 days post-infection in the hilus of the dentate gyrus. *n* = 4 control, *n* = 5 H1N1 mice.

(E) Quantification of gray matter reactive microglia (IBA1$^+$ CD68$^+$) 7 days post-infection in the M2 region of cortex. *n* = 5 mice per group.

(F) Quantification of white matter OPCs (PDGFRα^+) 7 days post-infection in the cingulum. *n* = 5 mice per group.

(G) Quantification of gray matter OPCs (PDGFRα^+) 7 days post-infection in the M2 region of cortex. *n* = 5 mice per group.

(H) Quantification of gray matter mature oligodendrocytes (ASPA$^+$) 7 days post-infection in the M2 region of cortex. *n* = 5 mice per group.

Data shown as mean ± SEM; each dot represents an individual mouse. ns: $p > 0.05$, * $p < 0.05$, analyzed via unpaired two-tailed t-test. (D) Scale bars, 40 μm.

bioRxiv preprint doi: https://doi.org/10.64898/2026.08.08.743593; this version posted August 13, 2026. The copyright holder for this preprint (which was not certified by peer review) is the author/funder, who has granted bioRxiv a license to display the preprint in perpetuity. It is made available under a CC-BY 4.0 International license.

1132 **Figure S3**

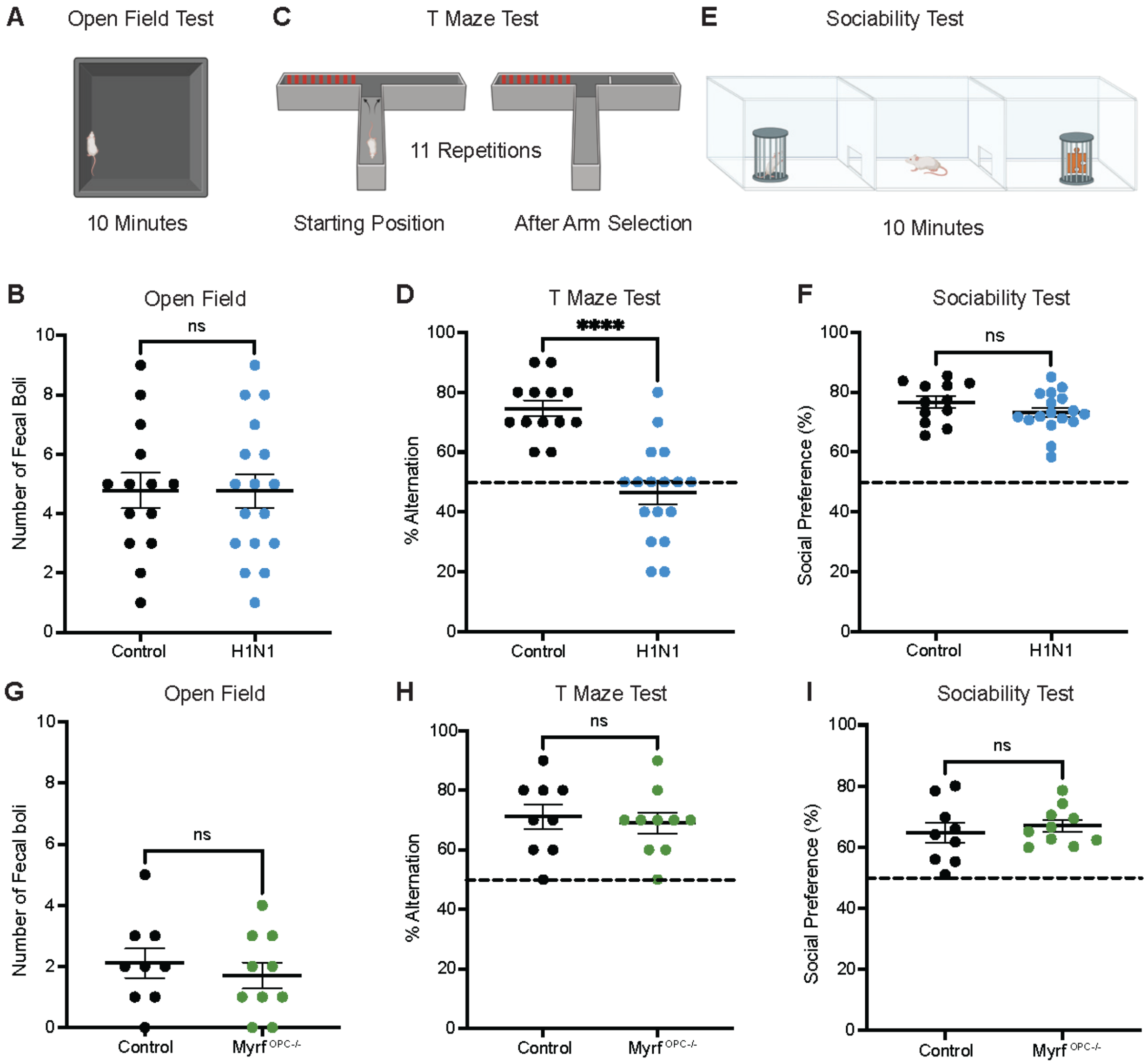

1133

bioRxiv preprint doi: https://doi.org/10.64898/2026.08.08.743593; this version posted August 13, 2026. The copyright holder for this preprint (which was not certified by peer review) is the author/funder, who has granted bioRxiv a license to display the preprint in perpetuity. It is made available under a CC-BY 4.0 International license.

Figure S3. Hippocampal dependent cognitive deficits observed at four weeks post-infection, related to **Figure 2**.

(A) Schematic illustration of the open field to assess anxiety-like behavior. Number of fecal boli in arena was quantified following a 10-minute testing period.

(B) Schematic illustration of spontaneous T-maze alternation test to assess spatial working memory and hippocampal-related cognitive dysfunction. Percent alternation is calculated over 11 trials, allowing for 10 decisions.

(C) Schematic illustration of three-chamber sociability test to assess social behavior and preference for social interaction. Social preference is defined as the percentage of time spent interacting with the conspecific mouse relative to total time spent interacting with either mouse or object.

(D) Total number of fecal boli in the open field over 10-minute testing period. Open field performed at 4 weeks post-infection. n = 14 control, n = 17 H1N1 mice.

(E) Percentage of alternations in T-maze performed at 4 weeks post-infection. n = 13 control, n = 17 H1N1 mice.

(F) Sociability test performed at 4 weeks post-infection over 10-minute testing period. n = 12 control, n = 17 H1N1 mice.

(G) Total number of fecal boli in the open field over 10-minute testing period. Open field performed at P42, 4 weeks post-injection. n = 7 control, n = 6 $Myrf^{OPC-/-}$ mice.

(H) Percentage of alternations in T-maze performed at P42, 4 weeks post-injection. n = 7 control, n = 6 $Myrf^{OPC-/-}$ mice.

(I) Sociability test performed at 4 weeks post-infection over 10-minute testing period. n = 7 control, n = 6 $Myrf^{OPC-/-}$ mice.

Data shown as mean ± SEM; each dot represents an individual mouse. ns: $p > 0.05$, **** $p < 0.0001$, analyzed via (D-G, I) unpaired two-tailed t-test or (B and H) Mann-Whitney U test for datasets that failed the Shapiro-Wilk test.

bioRxiv preprint doi: https://doi.org/10.64898/2026.08.08.743593; this version posted August 13, 2026. The copyright holder for this preprint (which was not certified by peer review) is the author/funder, who has granted bioRxiv a license to display the preprint in perpetuity. It is made available under a CC-BY 4.0 International license.

1178 **Figure S4**

1179

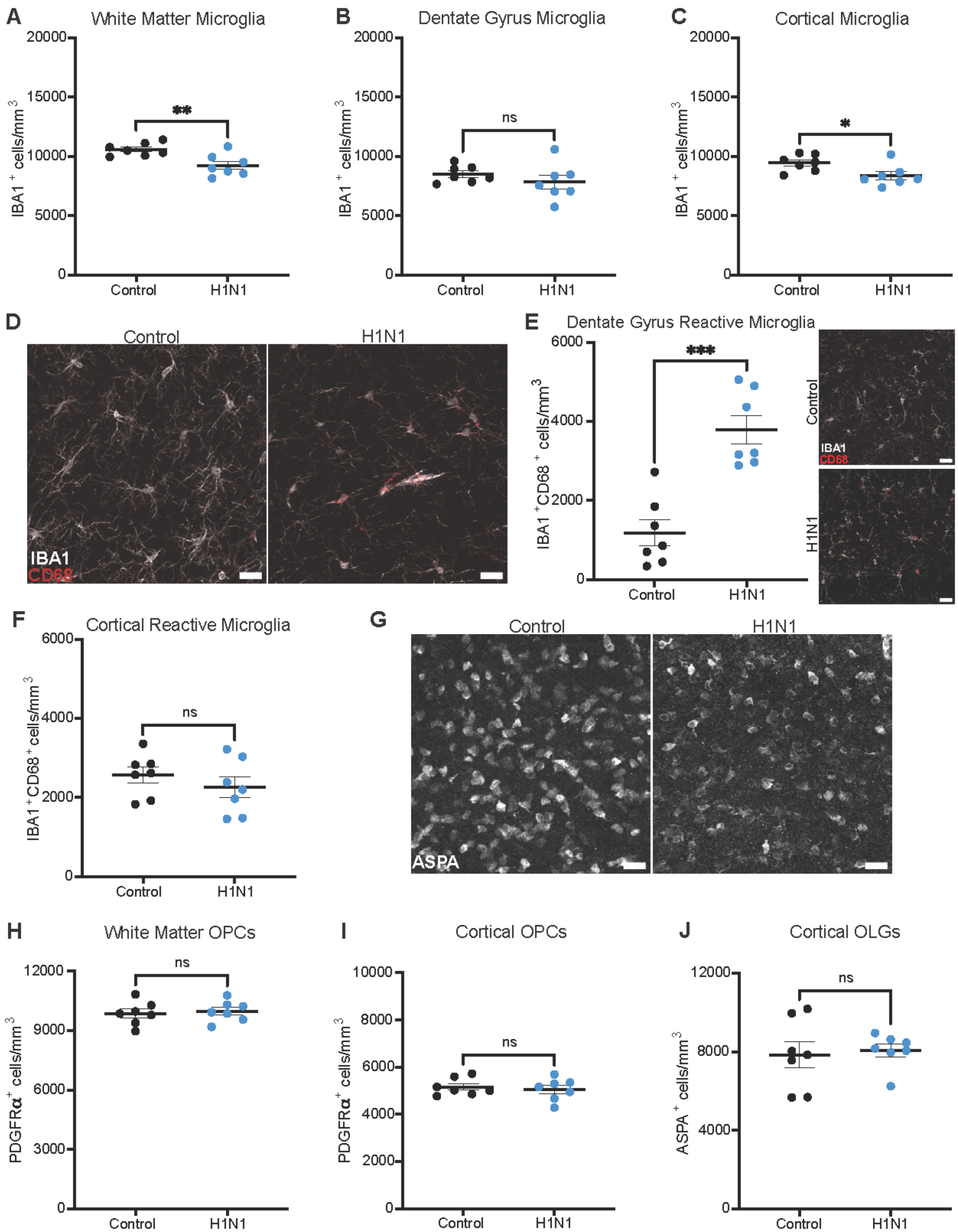

1180

bioRxiv preprint doi: https://doi.org/10.64898/2026.08.08.743593; this version posted August 13, 2026. The copyright holder for this preprint (which was not certified by peer review) is the author/funder, who has granted bioRxiv a license to display the preprint in perpetuity. It is made available under a CC-BY 4.0 International license.

Figure S4. Glial cell counts 4 weeks post-infection in the white matter, dentate gyrus, and cortex, related to **Figure 2**.

(A) Quantification of white matter microglia (IBA1$^+$) 4 weeks post-infection in the cingulum. *n* = 7 mice per group.

(B) Quantification of white matter microglia (IBA1$^+$) 4 weeks post-infection in the hilus of the dentate gyrus. *n* = 7 mice per group.

(C) Quantification of gray matter microglia (IBA1$^+$) 4 weeks post-infection in the M2 region of cortex. *n* = 7 mice per group.

(D) Representative confocal micrographs of white matter reactive microglia (IBA1, white; CD68, red) in the cingulum 4 weeks post-infection.

(E) Quantification and representative confocal micrographs of white matter reactive microglia (IBA1$^+$CD68$^+$) 4 weeks post-infection in the hilus of the dentate gyrus. *n* = 7 mice per group.

(F) Quantification of gray matter reactive microglia (IBA1$^+$ CD68$^+$) 4 weeks post-infection in the M2 region of cortex. *n* = 7 mice per group.

(G) Representative confocal micrographs of white matter mature oligodendrocytes (ASPA, white) in the cingulum 4 weeks post-infection.

(H) Quantification of white matter OPCs (PDGFRα^+) 4 weeks post-infection in the cingulum. *n* = 7 mice per group.

(I) Quantification of gray matter OPCs (PDGFRα^+) 4 weeks post-infection in the M2 region of cortex. *n* = 7 mice per group.

(J) Quantification of gray matter mature oligodendrocytes (ASPA$^+$) 4 weeks post-infection in the M2 region of cortex. *n* = 7 mice per group.

Data shown as mean ± SEM; each dot represents an individual mouse. ns: $p > 0.05$, * $p < 0.05$, ** $p < 0.01$, *** $p < 0.001$, analyzed via unpaired two-tailed t-test. (D, E, and G) Scale bars, 40 μm.

bioRxiv preprint doi: https://doi.org/10.64898/2026.08.08.743593; this version posted August 13, 2026. The copyright holder for this preprint (which was not certified by peer review) is the author/funder, who has granted bioRxiv a license to display the preprint in perpetuity. It is made available under a CC-BY 4.0 International license.

1225 **Figure S5**

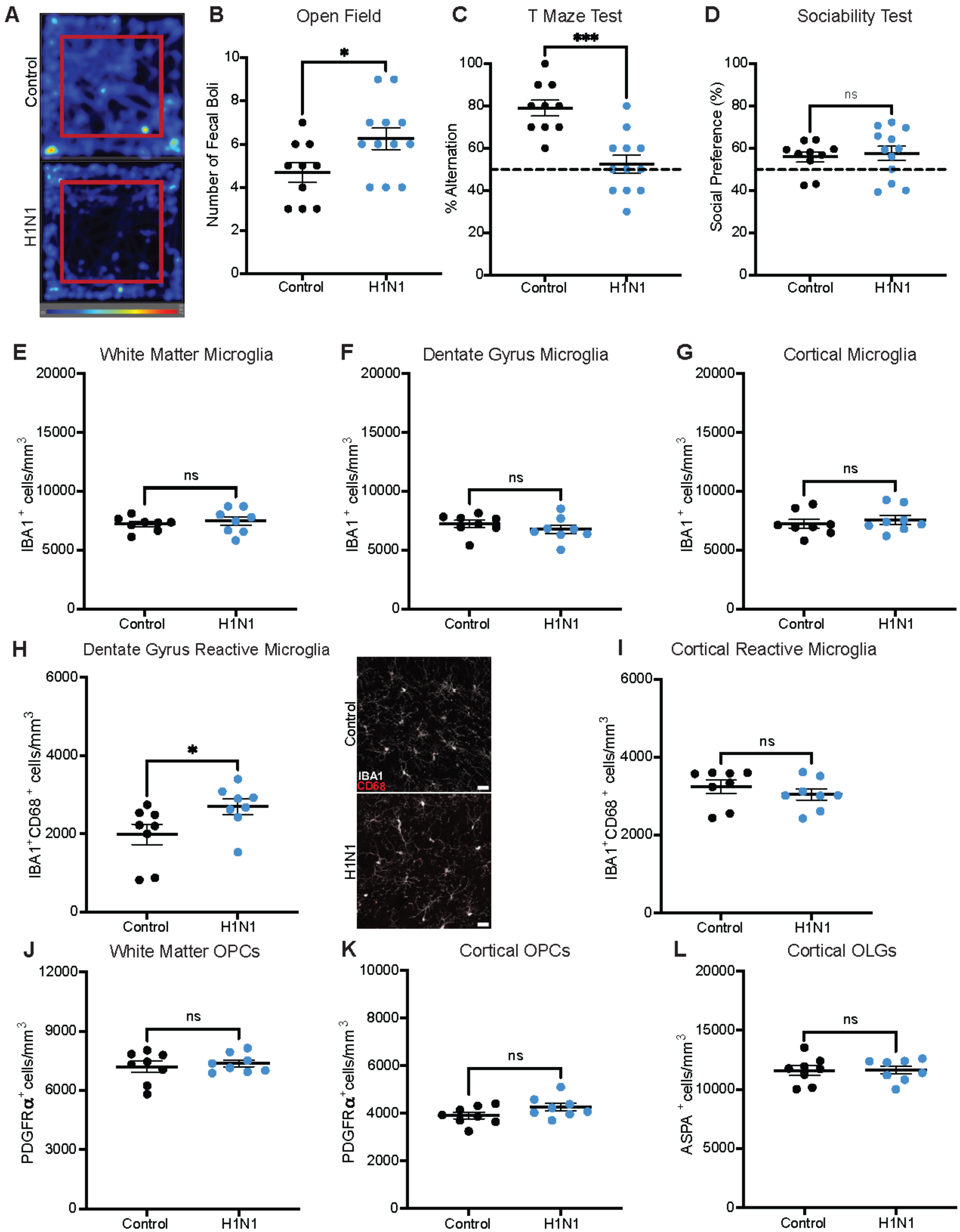

1226

bioRxiv preprint doi: https://doi.org/10.64898/2026.08.08.743593; this version posted August 13, 2026. The copyright holder for this preprint (which was not certified by peer review) is the author/funder, who has granted bioRxiv a license to display the preprint in perpetuity. It is made available under a CC-BY 4.0 International license.

Figure S5. Impact of juvenile H1N1 infection on glia and behavior at 8 weeks, related to **Figure 3**.

(A) Representative heatmaps illustrating time spent in the center (red square) and periphery of the arena during the open field test, with color intensity normalized across all trials (one mouse per trial).

(B) Total number of fecal boli in the open field over 10-minute testing period. Open field performed at 8 weeks post-infection. *n* = 10 control, *n* = 12 H1N1 mice.

(C) Percentage of alternations in T-maze performed at 8 weeks post-infection. *n* = 10 control, *n* = 12 H1N1 mice.

(D) Sociability test performed over 10-minute testing period at 8 weeks post-infection. *n* = 10 control, *n* = 12 H1N1 mice.

(E) Quantification of white matter microglia ($IBA1^+$) 8 weeks post-infection in the cingulum. *n* = 8 mice per group.

(F) Quantification of white matter microglia ($IBA1^+$) 8 weeks post-infection in the hilus of the dentate gyrus. *n* = 8 mice per group.

(G) Quantification of cortical gray matter microglia ($IBA1^+$) 8 weeks post-infection in the M2 region of cortex. *n* = 8 mice per group.

(H) Quantification and representative confocal micrographs of white matter reactive microglia ($IBA1^+CD68^+$) 8 weeks post-infection in the hilus of the dentate gyrus. *n* = 8 mice per group.

(I) Quantification of cortical gray matter reactive microglia ($IBA1^+$ $CD68^+$) 8 weeks post-infection in the M2 region of cortex. *n* = 8 mice per group.

(J) Quantification of white matter OPCs ($PDGFR\alpha^+$) 8 weeks post-infection in the cingulum. *n* = 8 mice per group.

(K) Quantification of gray matter OPCs ($PDGFR\alpha^+$) 8 weeks post-infection in the M2 region of cortex. *n* = 8 mice per group.

(L) Quantification of gray matter mature oligodendrocytes ($ASPA^+$) 8 weeks post-infection in the M2 region of cortex. *n* = 8 mice per group.

bioRxiv preprint doi: https://doi.org/10.64898/2026.08.08.743593; this version posted August 13, 2026. The copyright holder for this preprint (which was not certified by peer review) is the author/funder, who has granted bioRxiv a license to display the preprint in perpetuity. It is made available under a CC-BY 4.0 International license.

Data shown as mean ± SEM; each dot represents an individual mouse. ns: $p > 0.05$, * $p < 0.05$, *** $p < 0.001$, analyzed via (B, C, E-H, J-L) unpaired two-tailed t-test or (D and I) Mann-Whitney U test for datasets that failed the Shapiro-Wilk test. (G) Scale bars, 40 μm.

bioRxiv preprint doi: https://doi.org/10.64898/2026.08.08.743593; this version posted August 13, 2026. The copyright holder for this preprint (which was not certified by peer review) is the author/funder, who has granted bioRxiv a license to display the preprint in perpetuity. It is made available under a CC-BY 4.0 International license.

Figure S6

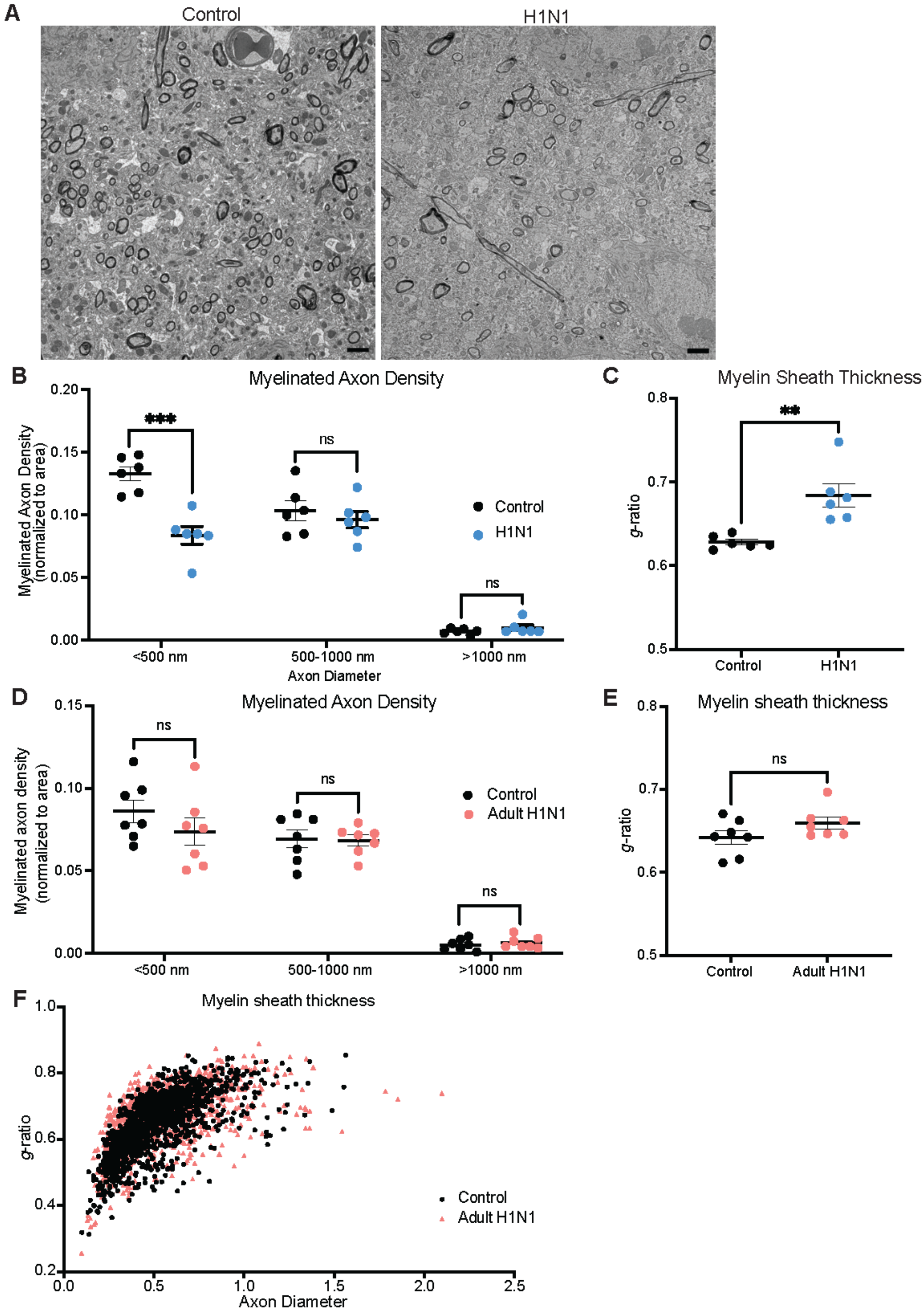

bioRxiv preprint doi: https://doi.org/10.64898/2026.08.08.743593; this version posted August 13, 2026. The copyright holder for this preprint (which was not certified by peer review) is the author/funder, who has granted bioRxiv a license to display the preprint in perpetuity. It is made available under a CC-BY 4.0 International license.

Figure S6. Juvenile H1N1 infection causes a decrease in myelinated axon density specifically in small diameter axons, related to **Figure 3**.

(A) Representative TEM images at the level of the cingulum of the corpus callosum in cross-section in mice infected during the juvenile period. Myelinated axons visible as electron-dense sheaths encircling axons.

(B) Myelinated axon density relative to small (<500 nm), medium (500-1000 nm), and large (>1000 nm) caliber axons for control (black points) or H1N1 (blue points) treated mice infected during the juvenile period 8 weeks post-infection. *n* = 6 mice per group.

(C) Quantification of *g*-ratio of mice infected during the juvenile period 8 weeks post-infection. *n* = 6 mice per group.

(D) Myelinated axon density relative to small (<500 nm), medium (500-1000 nm), and large (>1000 nm) caliber axons for control (black points) or H1N1 (orange points) treated mice injected during adulthood. *n* = 6 mice per group.

(E) Quantification of *g*-ratio of mice infected during the juvenile period. *n* = 7 mice per group.

(F) Scatter plots of *g*-ratio as a function of axon diameter 8 weeks post-infection for control axons (black dots) or H1N1 axons (orange triangles). Mice were infected during adulthood. A single point indicates the *g*-ratio for a single axon. Approximately 150-250 axons were quantified for each animal. *n* = 7 mice per group.

Data shown as mean ± SEM; each dot represents an individual mouse. ns: $p > 0.05$, ** $p < 0.01$, *** $p < 0.00$, analyzed via (B) unpaired two-tailed t-test with correction for multiple comparisons or (C) unpaired two-tailed t-test. (A) Scale bars, 2 μm.

bioRxiv preprint doi: https://doi.org/10.64898/2026.08.08.743593; this version posted August 13, 2026. The copyright holder for this preprint (which was not certified by peer review) is the author/funder, who has granted bioRxiv a license to display the preprint in perpetuity. It is made available under a CC-BY 4.0 International license.

Figure S7

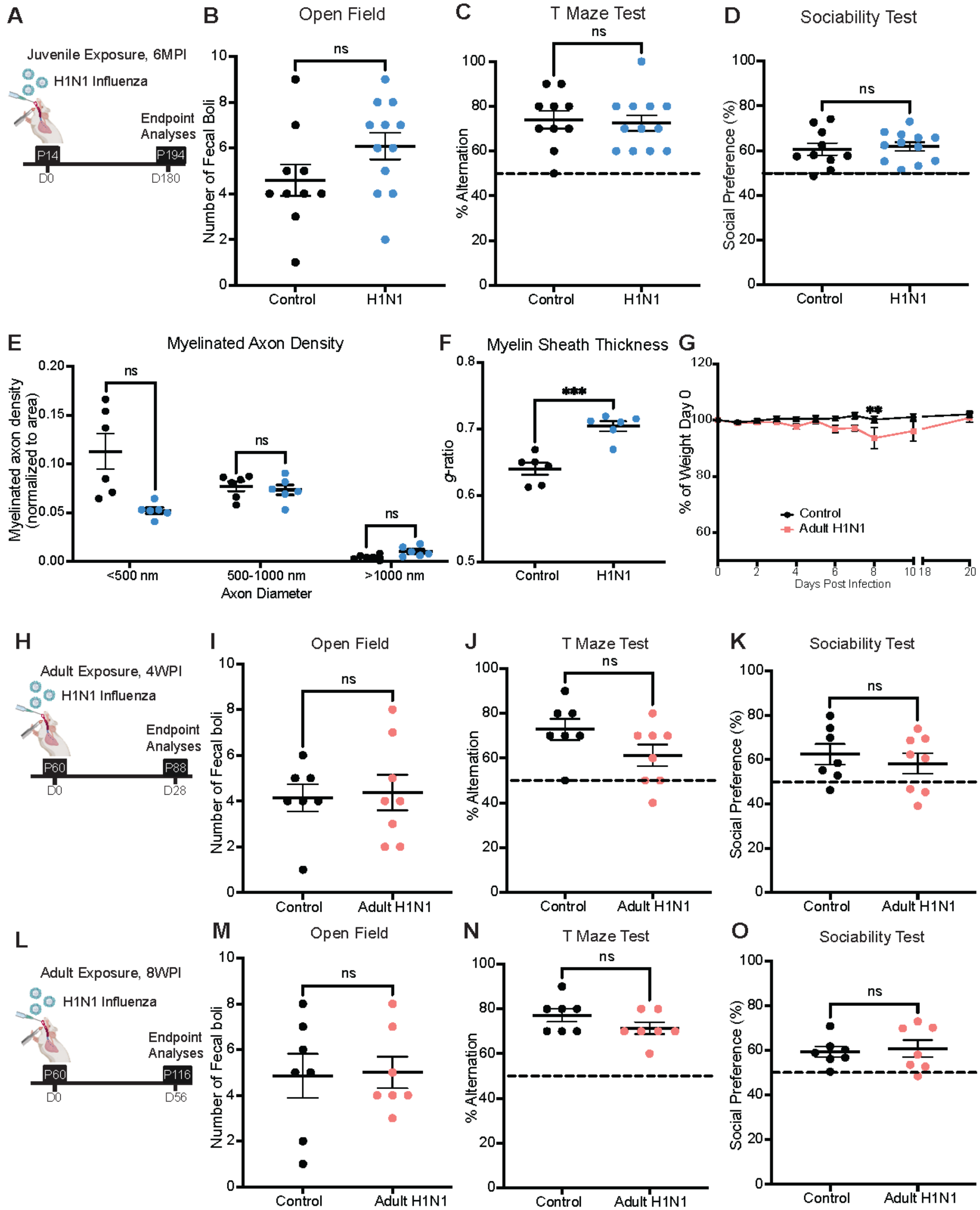

bioRxiv preprint doi: https://doi.org/10.64898/2026.08.08.743593; this version posted August 13, 2026. The copyright holder for this preprint (which was not certified by peer review) is the author/funder, who has granted bioRxiv a license to display the preprint in perpetuity. It is made available under a CC-BY 4.0 International license.

Figure S7. Behavioral assessments of anxiety-like behavior, sociability, and hippocampal function at chronic timepoints following juvenile and adult H1N1 infection, related to **Figure 4**.

(A) Schematic illustration of experimental timeline. Mice were intratracheally inoculated at P14 with PBS or H1N, and endpoint analyses were performed at P194, 6 months post-infection (MPI).

(B) Total number of fecal boli in the open field over 10-minute testing period. Open field performed at 6 months post-infection. *n* = 10 control, *n* = 12 H1N1 mice.

(C) Percentage of alternations in T-maze performed at 6 months post-infection. *n* = 10 control, *n* = 12 H1N1 mice.

(D) Sociability test performed over 10-minute testing period at 6 months post-infection. *n* = 10 control, *n* = 12 H1N1 mice.

(E) Myelinated axon density relative to small (<500 nm), medium (500-1000 nm), and large (>1000 nm) caliber axons for control (black points) or H1N1 (blue points) treated mice infected during the juvenile period 6 months post-infection. *n* = 6 mice per group.

(F) Quantification of *g*-ratio of mice infected during the juvenile period 6 months post-infection. *n* = 6 mice per group.

(G) Body weight (% of day 0 weight) of control and H1N1 influenza mice infected during adulthood. *n* = 7-10 mice per group per timepoint.

(H) Schematic illustration of experimental timeline. Mice were intratracheally inoculated at P60 with PBS or H1N1, and endpoint analyses were performed at P88, 4 weeks post-infection.

(I) Total number of fecal boli in the open field. Open field performed at 4 weeks post-infection. *n* = 7 control, *n* = 8 H1N1 mice.

(J) Percentage of alternations in T-maze performed at 4 weeks post-infection. *n* = 7 control, *n* = 8 H1N1 mice.

(K) Sociability test performed at 4 weeks post-infection. *n* = 7 control, *n* = 8 H1N1 mice.

(L) Schematic illustration of experimental timeline. Mice were intratracheally inoculated at P60 with PBS or H1N1, and endpoint analyses performed at P116, 8 weeks post-infection.

(M) Total number of fecal boli in the open field. Open field performed at 8 weeks post-infection. *n* = 7 mice per group.

bioRxiv preprint doi: https://doi.org/10.64898/2026.08.08.743593; this version posted August 13, 2026. The copyright holder for this preprint (which was not certified by peer review) is the author/funder, who has granted bioRxiv a license to display the preprint in perpetuity. It is made available under a CC-BY 4.0 International license.

(N) T-maze performed at 8 weeks post-infection. $n = 7$ mice per group.

(O) Sociability test performed at 8 weeks post-infection. $n = 7$ mice per group.

Data shown as mean ± SEM; each dot represents an individual mouse. ns: $p > 0.05$, analyzed via (B, D, F, I-K, M-O) unpaired two-tailed t-test, (C) Mann-Whitney U test for datasets that failed the Shapiro-Wilk test, or (E) unpaired two-tailed t-test with correction for multiple comparisons, or (G) two-way ANOVA with multiple comparisons.

bioRxiv preprint doi: https://doi.org/10.64898/2026.08.08.743593; this version posted August 13, 2026. The copyright holder for this preprint (which was not certified by peer review) is the author/funder, who has granted bioRxiv a license to display the preprint in perpetuity. It is made available under a CC-BY 4.0 International license.

Figure S8

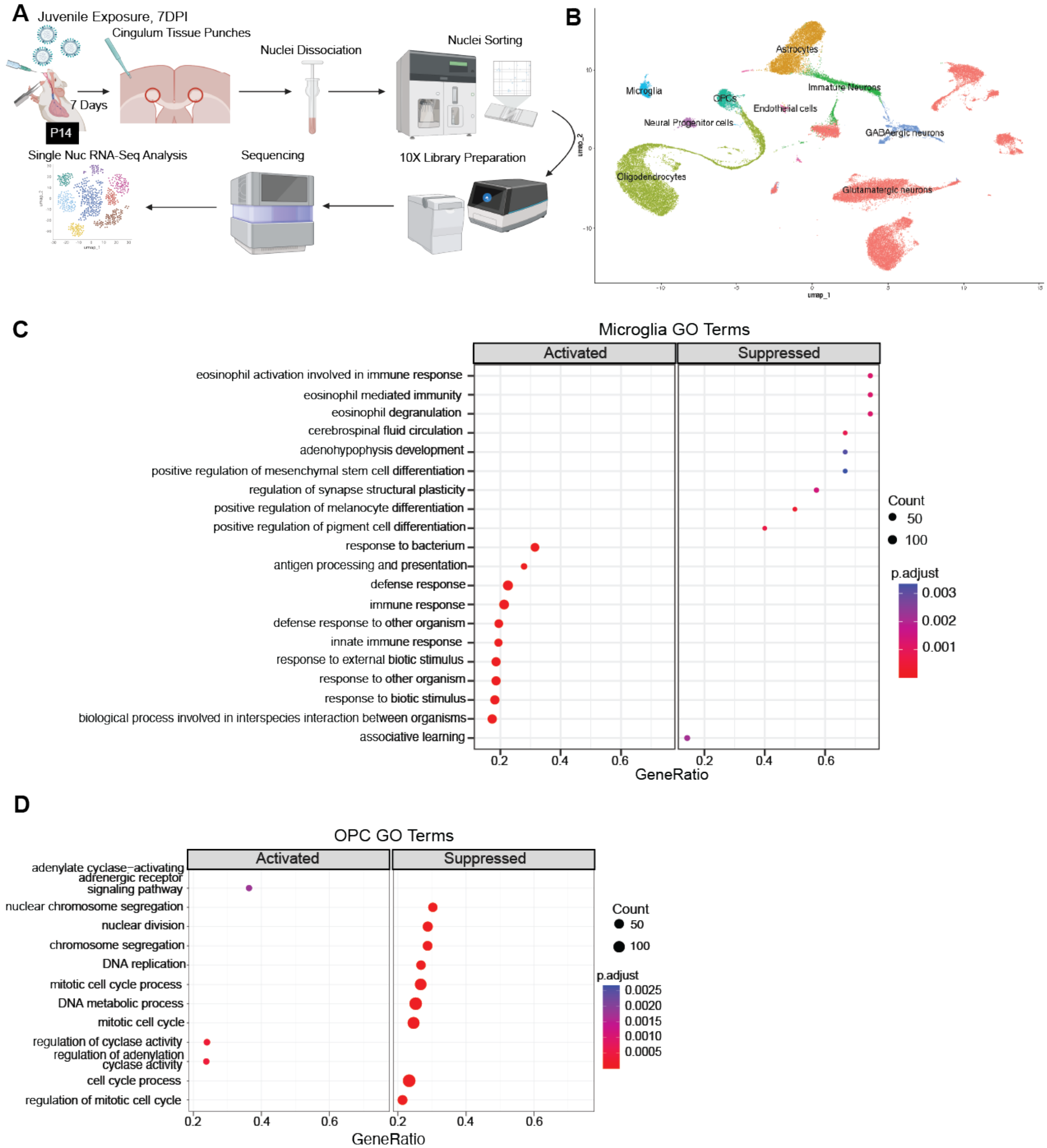

bioRxiv preprint doi: https://doi.org/10.64898/2026.08.08.743593; this version posted August 13, 2026. The copyright holder for this preprint (which was not certified by peer review) is the author/funder, who has granted bioRxiv a license to display the preprint in perpetuity. It is made available under a CC-BY 4.0 International license.

Figure S8. H1N1 alters transcriptional profiles of microglia and OPCs in the cingulum, related to **Figure 5**.

(A) Schematic of experimental paradigm, sample collection and processing. Mice were intratracheally inoculated with PBS or H1N1 at P14, and brains were collected 7 days post-infection. Cingulum tissue punches were collected, nuclei were dissociated and sorted, and then libraries were prepared to perform single-nucleus RNA sequencing and analysis. n = 3 mice per group.

(B) UMAP plot of 54,226 single nuclei from the cingulum, divided between control and H1N1 samples (26,653 single nuclei from control mice and 27,573 single nuclei from H1N1 mice).

(C) Gene ontology (GO) enrichment analysis of differentially expressed genes in microglia cluster of H1N1 compared to control samples. Bubble plot displays the top enriched biological process (BP) GO terms for marker genes identified via single-nucleus RNA sequencing, The x-axis indicates the number of genes associated with the GO term divided by the total number of genes in the cluster. The bubble size is proportional to the number of genes enriched in each GO term, while the color represents the adjusted p-value.

(D) GO enrichment analysis of differentially expressed genes in OPC cluster of H1N1 compared to control samples.

bioRxiv preprint doi: https://doi.org/10.64898/2026.08.08.743593; this version posted August 13, 2026. The copyright holder for this preprint (which was not certified by peer review) is the author/funder, who has granted bioRxiv a license to display the preprint in perpetuity. It is made available under a CC-BY 4.0 International license.

1501 **Figure S9**

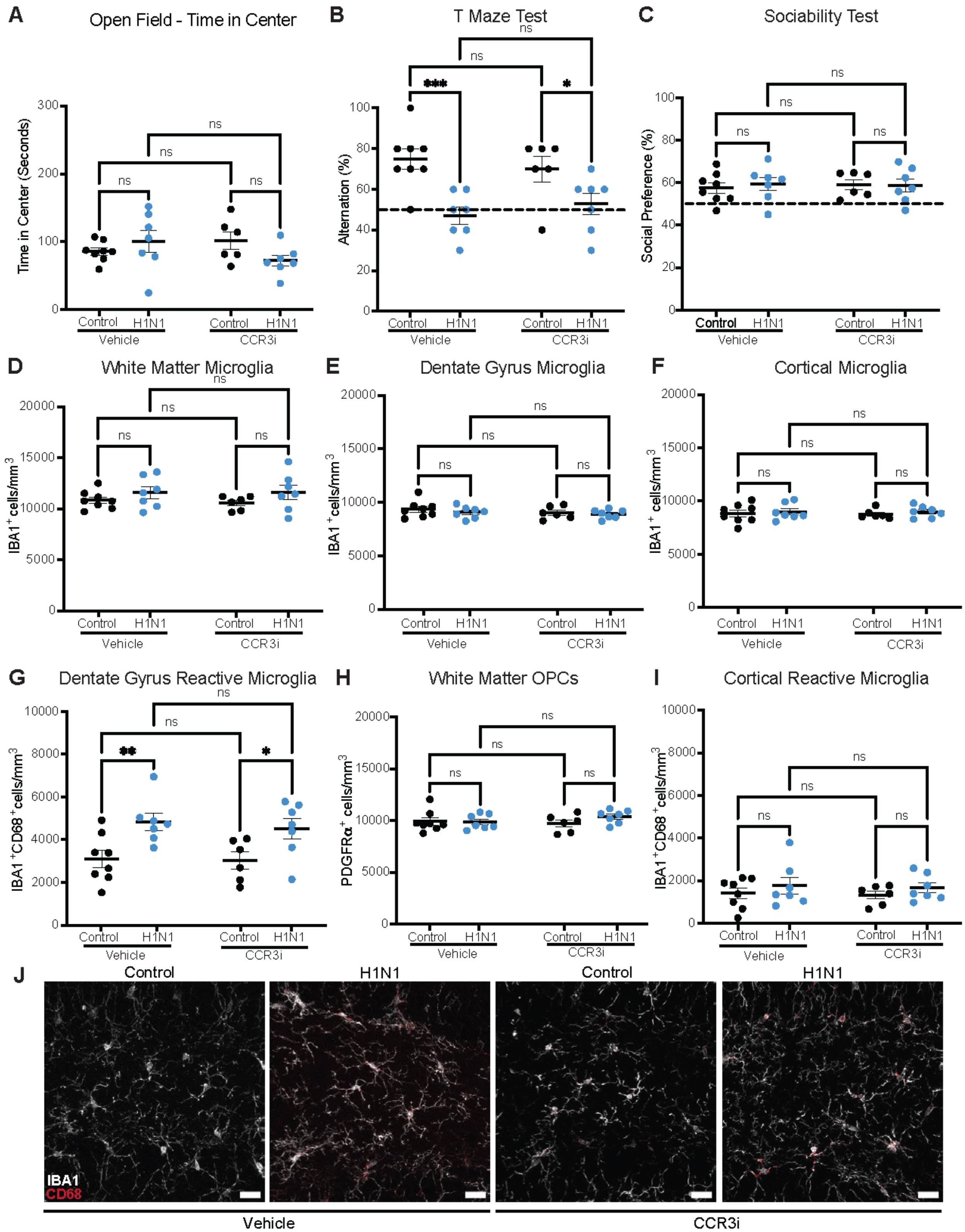

1502
1503

bioRxiv preprint doi: https://doi.org/10.64898/2026.08.08.743593; this version posted August 13, 2026. The copyright holder for this preprint (which was not certified by peer review) is the author/funder, who has granted bioRxiv a license to display the preprint in perpetuity. It is made available under a CC-BY 4.0 International license.

Figure S9. Chemokine receptor inhibition does not rescue hippocampal microglial reactivity or deficits in spontaneous alternating T-maze, related to **Figure 6**.

(A) Time spent in center of the open field over 10-minute testing period. *n* = 8, 7, 6, and 7 mice per group.

(B) Percentage of alternations in T-maze over 11 trials. *n* = 8, 7, 6, and 7 mice per group.

(C) Results for sociability test performed over 10-minute testing period. *n* = 8, 7, 6, and 7 mice per group.

(D) Quantification of white matter microglia ($IBA1^+$) in the cingulum. *n* = 8, 7, 6, and 7 mice per group.

(E) Quantification of white matter microglia ($IBA1^+$) in the hilus of the dentate gyrus. *n* = 8, 7, 6, and 7 mice per group.

(F) Quantification of gray matter microglia ($IBA1^+$) in the M2 region of cortex. *n* = 8, 7, 6, and 7 mice per group.

(G) Quantification of white matter OPCs ($PDGFR\alpha^+$) in the cingulum. *n* = 8, 7, 6, and 7 mice per group.

(H) Quantification of white matter reactive microglia ($IBA1^+CD68^+$) in the hilus of the dentate gyrus. *n* = 8, 7, 6, and 7 mice per group.

(I) Quantification of gray matter reactive microglia ($IBA1^+$ $CD68^+$) in the M2 region of cortex. *n* = 8, 7, 6, and 7 mice per group.

(J) Representative confocal micrographs of reactive microglia (IBA1, white; CD68, red) in the hilus of the dentate gyrus.

Data shown as mean ± SEM; each dot represents an individual mouse. ns: $p > 0.05$, * $p < 0.05$, ** $p < 0.01$, analyzed via two-way ANOVA. (J) Scale bars, 40 μm.
